# A comprehensive exploration of astrocytes in migraine: a bibliometric and visual analysis

**DOI:** 10.1186/s40001-024-01919-z

**Published:** 2024-06-10

**Authors:** Shijie Wei, Tianqi Du, Lili Zhang, Xuhao Li, Zhe Wang, Yike Ning, Yu Tang, Xinyu Wu, Jing Han

**Affiliations:** 1 School of Acupuncture and Tuina, Shandong University of Traditional Chinese Medicine, Jinan, China; 2 Center of Human Reproduction and Genetics, The Affiliated Suzhou Hospital of Nanjing Medical University, Suzhou, China; 3 Institute of Acupuncture and Moxibustion, Shandong University of Traditional Chinese Medicine, Jinan, China; 4 Affiliated Hospital of Shandong University of Traditional Chinese Medicine, Jinan, China

**Keywords:** Bibliometric analysis, Migraine, Astrocytes, Astroglias

## Abstract

**Background:**

Migraine, as a prevalent neurologic disorder, involves intricate and yet incompletely elucidated pathophysiological mechanisms. A plethora of research findings underscores the pivotal role played by astrocytes in the progression of migraines. In order to elucidate the current advances and directions in research pertaining to astrocytes in migraines, we conducted bibliometric analysis of relevant literature and visualized the results. Subsequently, we expound upon these findings to contribute to the evolving understanding of the role of astrocytes in migraine pathophysiology.

**Methods:**

On November 21, 2023, we conducted a search on Web of Science (WOS), restricting the document type to articles or reviews and language to English. Following a meticulous selection process involving three researchers, we identified the literature to be included in our analysis. Subsequently, we employed Microsoft Office Excel programs, R, VOSviewer, Scimago Graphica, and CiteSpace software to conduct visualization analysis of basic information and trends regarding journals, countries/regions, and influential authors, institutions, keywords, and papers.

**Results:**

As of November 21, 2023, relevant literature has been published in 71 journals across 27 countries/regions. This corpus comprises contributions from 576 authors affiliated with 220 institutions, encompassing 865 keywords and referencing 6065 scholarly articles. CEPHALALGIA stands out as the most influential journal in this field, while authors PIETROBON D and DALKARA T have significant impact. The United States is highly influential, with CNR and UNIV PADUA emerging as highly influential institutions. The predominant category is Neurosciences.

**Conclusions:**

Future investigators may continue to focus on migraines with aura, familial hemiplegic migraine (FHM), and the crucial calcitonin gene-related peptide (CGRP) system. Employing advanced observational techniques, such as imaging, researchers should pay attention to cellular and tissue structures, such as microglia and the trigeminal ganglion, as well as mechanisms involving inflammation and central sensitization. Moreover, animal models are paramount in obtaining high-quality evidence.

## Introduction

Migraine, a widespread and incapacitating neurological condition, impacts more than 15% of the world’s population [1]. Its primary clinical manifestations often include episodic, pulsatile, moderate-to-severe headaches, typically lasting 4–72 h. These headaches may be unilateral and accompanied by symptoms such as nausea, vomiting, photophobia, phonophobia, and exacerbation during routine physical activity. The classification of migraines is intricate, encompassing various subphenotypes, such as Migraine without aura, Migraine with aura, chronic migraine, and others [2]. Our current understanding of migraines remains limited, prompting extensive research endeavors to decipher the pathophysiological mechanisms underlying this condition. Among these, the trigeminovascular system has gained widespread recognition and attention [3]. In addition, various factors such as peripheral nerve compression [4], sex hormones [5], and genetic influences [6] are considered to exert significant, undeniable impacts on the progression of migraines. Astrocytes are common glial cells in the central nervous system (CNS) and play intricate roles in various functions and diseases within the CNS [7]. Current evidence indicates that astrocytes are involved in the initiation and propagation of Cortical Spreading Depression (CSD) associated with migraines [8]. Mutations in astrocyte-related Na + /K + -ATPase are a primary cause of FHM type 2 (FHM2) [9]. Furthermore, astrocytes in the migraine process exhibit intricate relationships with neurons and microglial cells, involving sites such as the trigeminal nucleus caudalis and trigeminal ganglia. They actively participate in complex mechanisms, including inflammation and central sensitization [10, 11]. In conclusion, astrocytes have emerged as pivotal contributors to the mechanisms of migraines. A thorough exploration of the role of astrocytes in migraines holds the potential to become a viable therapeutic avenue, alleviating the burden on migraine patients.

Over the past few decades, the crucial role of astrocytes in migraines has been gradually unveiled. However, there is a lack of comprehensive and objective assessments regarding publications, journals, authors, countries, and institutions in this field. The research trajectory and trends in this domain are currently lacking the necessary evaluations. Bibliometrics enables researchers to gain comprehensive insights into the landscape of research within a specific field by conducting thorough analyses of publications [12, 13]. However, we did not identify any bibliometric studies describing the role of astrocytes in migraines. Therefore, we employed bibliometric methods to analyze relevant literature within the field and visualize the findings. This enabled us to summarize the developmental trajectory of the field, explore current research hotspots, and provide a reasoned outlook for future trajectories. Our study will not only assist scholars in gaining a comprehensive overview of the field but will also contribute to scholars undertaking new investigations and making novel discoveries in this domain.

## Methods

### Data collection

Web of Science, renowned for its authority, representativeness, and widespread acceptance as a bibliographic database, furnishes comprehensive and precise literature records and abundant citation analysis data essential for this study [14]. Therefore, we conducted a comprehensive literature search on November 21, 2023, using the Science Citation Index Expanded (SCI-E) database from WOS (https://www.webofscience.com). The search formula used was: [TS = (Astrocytes) OR TS = (Astrocytic) OR TS = (Astrocytic Cell) OR TS = (Astrocytic Cells) OR TS = (Astrocyte) OR TS = (Astroglia) OR TS = (Astroglias) OR TS = (Astroglial Cells) OR TS = (Astroglial Cell) OR TS = (Cell, Astroglial) OR TS = (Astroglia Cells) OR TS = (Astroglia Cell) OR TS = (Cell, Astroglia) OR TS = (Star-shaped glial cells) OR TS = (Star shaped glial cells)) AND (TS = (Migraine Headaches) OR TS = (Migraine Headache) OR TS = (Hemicrania Migraines) OR TS = (Abdominal Migraine) OR TS = (Migraine Disorders) OR TS = (Migraine Disorders) OR TS = (Variant, Migraine) OR TS = (Disorders, Migraine) OR TS = (Headache, Sick) OR TS = (Migraine Variant) OR TS = (Migraine Syndrome, Cervical) OR TS = (Migraines, Acute Confusional) OR TS = (Hemicrania Migraine) OR TS = (Migraine Disorder) OR TS = (Headaches, Migraine) OR TS = (Migraine Variants) OR TS = (Migraine) OR TS = (Migraines, Abdominal) OR TS = (Migraine Syndromes, Cervical) OR TS = (Migraines) OR TS = (Disorder, Migraine) OR TS = (Headaches, Sick) OR TS = (Cervical Migraine Syndrome) OR TS = (Cervical Migraine Syndromes) OR TS = (Headache, Migraine) OR TS = (Migraine, Abdominal) OR TS = (Acute Confusional Migraine) OR TS = (Abdominal Migraines) OR TS = (Migraine, Acute Confusional) OR TS = (Variants, Migraine) OR TS = (Sick Headache) OR TS = (Acute Confusional Migraines) OR TS = (Migraine, Hemicrania) OR TS = (Status Migrainosus) OR TS = (Sick Headaches) OR TS = (Migraines, Hemicrania)]. We restricted the publication categories to peer-reviewed English-language articles and reviews to ensure a higher quality of literature included in the analysis.

### Inclusion and exclusion criteria

Two scholars independently conducted initial screening by reviewing the titles, abstracts, keywords, and full texts of papers, aiming to exclude literature unrelated to the topic. When two researchers encounter discrepancies regarding the inclusion/exclusion of articles, they seek the input of a third researcher to achieve consensus.

### Data analysis and visualization

This study primarily utilized four software programs for bibliometric analysis and visualization. In particular, Microsoft Office Excel 2021 was utilized for creating visualizations of Times Cited and Publications Over Time within the field, as well as for data summarization and table creation. VOSviewer (version 1.6.19) [15] was utilized to generate temporal evolution network visualizations for journals, countries/regions, influential authors, and institutions, and to contribute to the execution of co-occurrence and co-authorship analyses for journals, countries/regions, influential authors, and institutions. Scimago Graphica (version 1.0.35) [16] Scimago Graphica (version 1.0.35) [15] was utilized to generate visualizations depicting the co-occurrence and co-authorship patterns among journals, countries/regions, as well as influential authors and institutions. The R package ‘bibliometrix’ (version 4.3.1) (https://www.bibliometrix.org) [17] was utilized for historiographic analysis and journal/author publication trends over the years and for computing relevant metrics such as g-index, h-index, the number of citations (NC), and the number of publications (NP) for the included literature. CiteSpace (version 6.2.R4) [18] was utilized to generate the dual journal map focusing on journals, and to perform co-occurrence, burstiness analysis, and clustering analyses for references and keywords.

## Results

### Analysis of the overall publication status

**Fig. 1:**
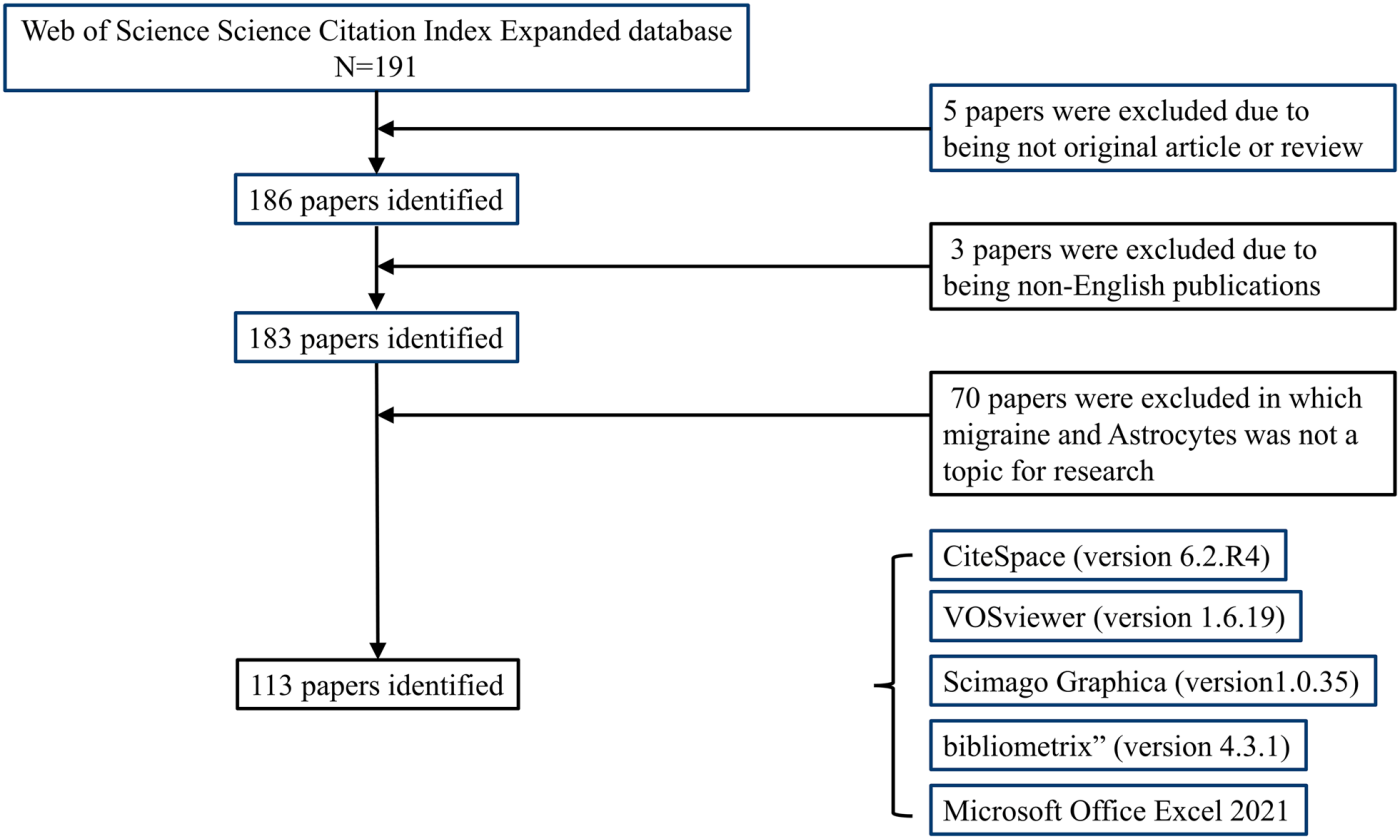
Flowchart depicting the literature screening process

The retrieval process, as depicted in Fig. 1, was conducted on November 21, 2023, resulting in a sum of 191 publications, comprising 186 articles and reviews. After excluding non-English publications, 183 remained. Subsequently, researchers applied inclusion and exclusion criteria, resulting in the final inclusion of 113 studies meeting the criteria. These publications spanned across 27 countries/regions, were sourced from 71 journals, involved 576 authors, and originated from 220 institutions. This comprehensive review underscores the global and interdisciplinary nature of the research landscape under consideration.

### Times cited and publications over time

**Fig. 2:**
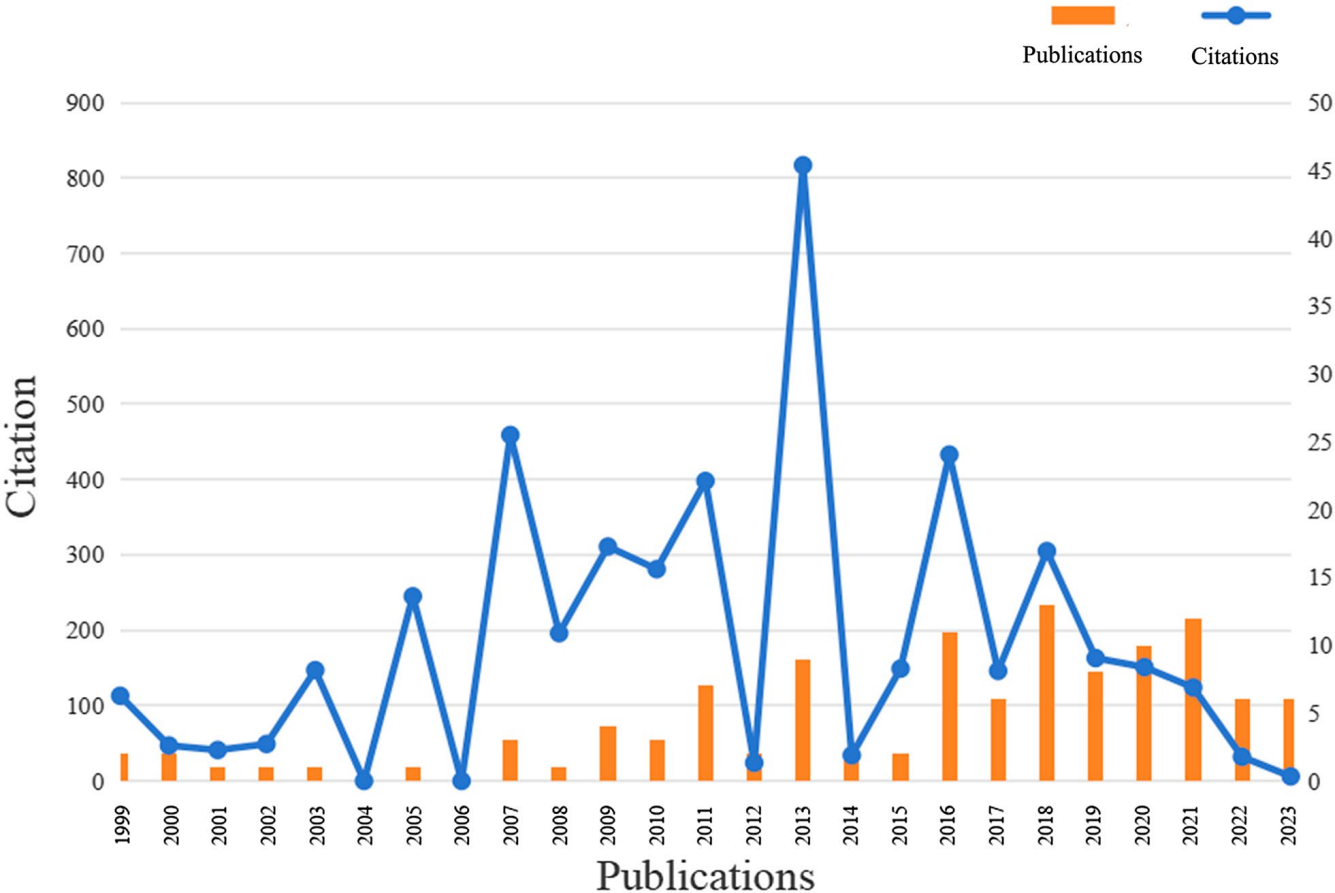
Timeline of publication and citation

We illustrate the Times Cited and Publications status of the relevant research (Fig. 2). The peak for Times Cited was reached in 2013, reaching its highest level at *N* = 817. In addition, in both 2007 (*N* = 459) and 2016 (*N* = 433), the number exceeded 400 citations. The trend in the number of Publications exhibits a consistent increase, reaching its peak in 2018 with a total of 13 publications.

### Article

The included publications analyzed have amassed a total of 3663 citations, with an average citation rate of 29.76 citations per article. Notably, 14 publications received more than 100 citations each. “Spreading depression triggers headache by activating neuronal Panx1 channels” stands out as the most cited publication, accumulating a total of 342 citations. Researchers have uncovered that CSD initiates the opening of neuronal Pannexin1 megachannels and the activation of caspase-1. This sequence of events leads to the release of high-mobility group box 1 from neurons and the activation of nuclear factor κB in astrocytes. Suppressing this cascade reaction has the potential to alleviate trigeminovascular activation induced by CSD [19]. Subsequently, the publication titled “Neuron–astrocyte interactions: partnership for normal function and disease in the central nervous system” emerges, accumulating a noteworthy 245 citations. In this work, the authors delve into the pivotal role of neuron–astrocyte interactions in the aura and episodes in migraine, as well as FHM [20]. The publication titled “Deficiency in Na,K-ATPase Alpha Isoform Genes Alters Spatial Learning, Motor Activity, and Anxiety in Mice” has garnered a total of 230 citations. The authors examined the impact of the Na,K-ATPase alpha 2 isoform, closely associated with FHM2 and predominantly expressed in astrocytes, on various behaviors in mice [21]. The aforementioned publications have garnered substantial attention, each accumulating over 200 citations, with a combined total exceeding 800 citations.

### Journal analysis

**Fig. 3:**
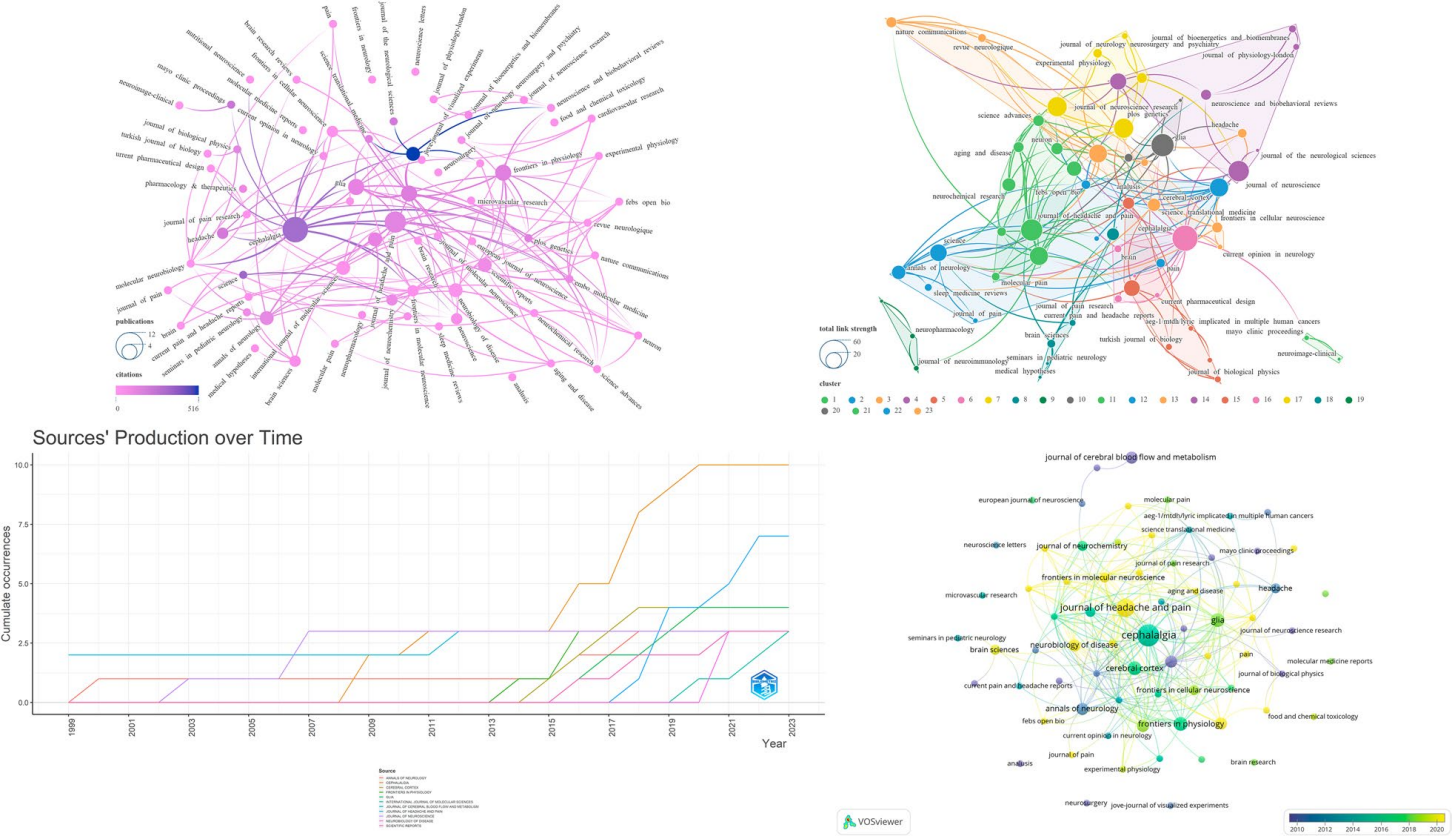
Visualization map illustrating journal contributions

Cluster analysis is a method of grouping targets where data within the same group exhibit high similarity, while data between different groups display low similarity [22]. The h-index is a widely used bibliometric indicator, wherein individuals with a greater number of publications and total citations tend to have higher h-index values [23]. Similar to the h-index, the g-index calculation places greater emphasis on reflecting the academic influence of highly cited items [24]. In addition, we downloaded the relevant data for the 2022 impact factor and QUARTILE of journals from the Journal Citation Reports (https://jcr.clarivate.com/jcr/browse-journals). We included all 71 journals related to the relevant literature in the analysis, followed by the creation of visualization maps. The analysis results are presented in Fig. 3. The journals included in the analysis were primarily categorized into 23 distinct clusters. Detailed data of the top 11 journals, sorted in descending order by NP, are listed in Table 1. Among the top 11 journals, over 70% are positioned within JIF Quartile 1. The journal ranked first in NP is CEPHALALGIA (*N* = 10), followed by JOURNAL OF HEADACHE AND PAIN (*N* = 7). The journal ranked first in NC are JOURNAL OF NEUROSCIENCE (*N* = 516) and CEPHALALGIA (*N* = 312). The journal ranked first in average citations is JOURNAL OF NEUROSCIENCE (*N* = 172), followed by CEREBRAL CORTEX (*N* = 41.5). The leading journals in both g-index and h-index standings are CEPHALALGIA (*N* = 10, *N* = 9) and JOURNAL OF HEADACHE AND PAIN (*N* = 7, *N* = 5). The journals ranked first and second in the Journal Impact Factor (JIF) are ANNALS OF NEUROLOGY (*N* = 11.2) and JOURNAL OF HEADACHE AND PAIN (*N* = 7.4), respectively.

**Table 1:** Leading 11 journals in terms of productivity

Rank	Journals	NP	NC	Total link strength	g index	h index	Average citations	JIF QUARTILE	JIF
1	Cephalalgia	10	312	47	10	9	31.2	Q1	4.9
2	Journal of headache and pain	7	134	34	7	5	19.14	Q1	7.4
3	Cerebral cortex	4	166	25	4	4	41.5	Q2	3.7
4	Frontiers in physiology	4	150	20	4	4	37.5	Q2	4
5	glia	4	122	37	4	4	30.5	Q1	6.2
6	Annals of neurology	3	107	14	3	3	35.67	Q1	11.2
7	Journal of cerebral blood flow and metabolism	3	122	1	3	3	40.67	Q1	6.3
8	Journal of neuroscience	3	516	31	3	3	172	Q1	5.3
9	Neurobiology of disease	3	24	16	3	3	8	Q1	6.1
10	Scientific reports	3	80	28	3	3	26.67	Q2	4.6
11	International journal of molecular sciences	3	16	10	3	2	5.33	Q1	6.5

Regarding the total link strength, CEPHALALGIA (*N* = 47) secures the top position, followed by GLIA (*N* = 37) and JOURNAL OF HEADACHE AND PAIN (*N* = 34).

**Fig. 4:**
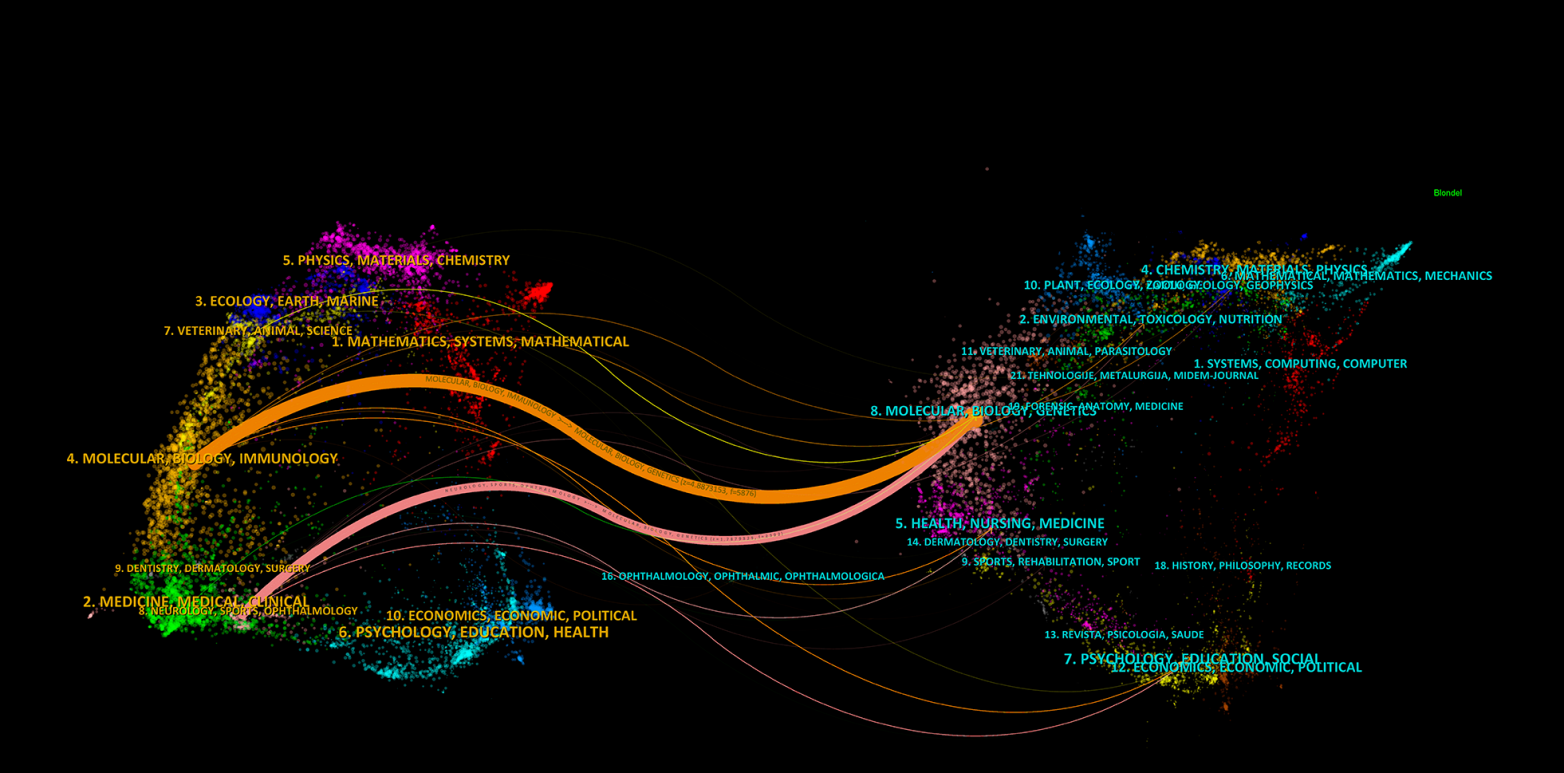
Dual map visualization of journals

The dual journal map provides a clear and intuitive depiction of citation relationships between journals and co-cited journals. The dual journal map consists of two parts: the citing map and the cited map, located respectively on the left and right sides of the dual journal map. The connecting lines in the middle of the dual journal map represent the citation relationships between journals and co-cited journals [25]. As illustrated in Fig. 4, citing literature from the field of MOLECULAR/BIOLOGY/IMMUNOLOGY and NEUROLOGY/SPORTS/OPHTHALMOLOGY primarily cites references from the field of MOLECUALR/BIOLOGY/GENETICS through two pathways. This demonstrates the primary citation relationships among journals.

### Author analysis

**Fig. 5:**
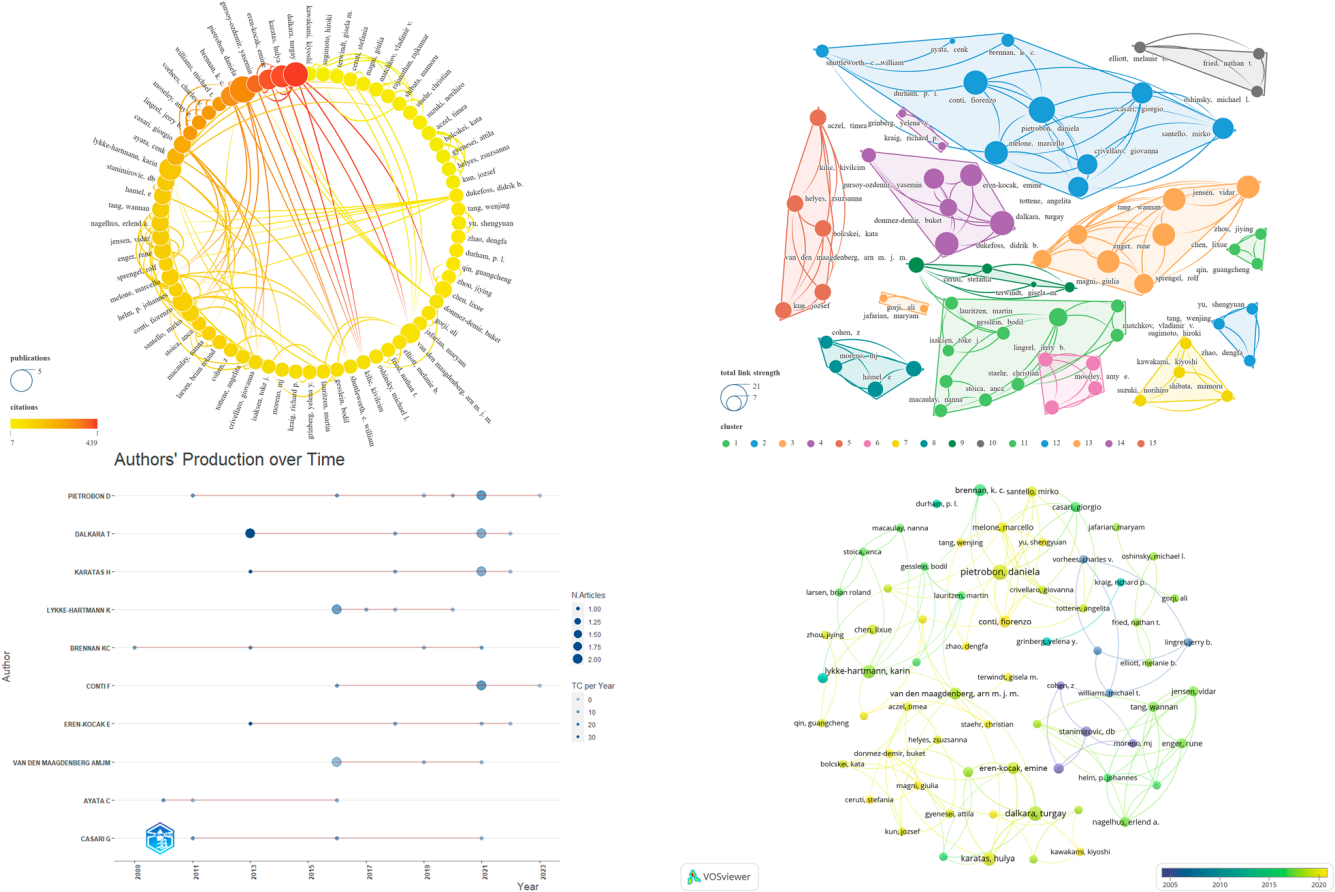
Visualization map illustrating Authors contributions

After consolidating synonymous author names, such as chen, li-xue and chen, lixue, we have identified a total of 576 authors involved in this study. We filtered out 68 authors who met the criteria of “Minimum number of documents of an article = 2” and conducted a visualization analysis on them (Fig. 5). The results revealed that these authors were divided into 15 main clusters, with detailed data of the top 8 authors, sorted in descending order by NP, listed in Table 2. The author ranked first in NP is PIETROBON D (*N* = 7), followed by DALKARA T (*N* = 6). Regarding NC scores, the top author is DALKARA T (*N* = 439), followed by KARATAS H (*N* = 412). The author ranked first in average citations is EREN-KOCAK E (*N* = 101.25), followed by KARATAS H (*N* = 82.40). The author ranked first in h-index is PIETROBON D (*N* = 6), followed by DALKARA T (*N* = 5) and LYKKE-HARTMANN K (*N* = 5). The author ranked first in g-index are PIETROBON D (*N* = 7) and DALKARA T (*N* = 6). Regarding the total link strength, PIETROBON D (*N* = 38) ranks first, followed by LYKKE-HARTMANN K (*N* = 35).

**Table 2:** Leading eight authors in terms of productivity

Rank	Authors	NP	NC	total link strength	g_index	h_index	Average citations
1	PIETROBON D	7	326	38	7	6	46.57
2	DALKARA T	6	439	32	6	5	73.17
3	KARATAS H	5	412	31	5	4	82.40
4	LYKKE-HARTMANN K	5	161	35	5	5	32.20
5	BRENNAN KC	4	315	23	4	4	78.75
6	CONTI F	4	127	27	4	3	31.75
7	EREN-KOCAK E	4	405	25	4	4	101.25
8	VAN DEN MAAGDENBERG AMJM	4	60	29	4	4	15.00

### Country/region and institution analysis

**Fig. 6:**
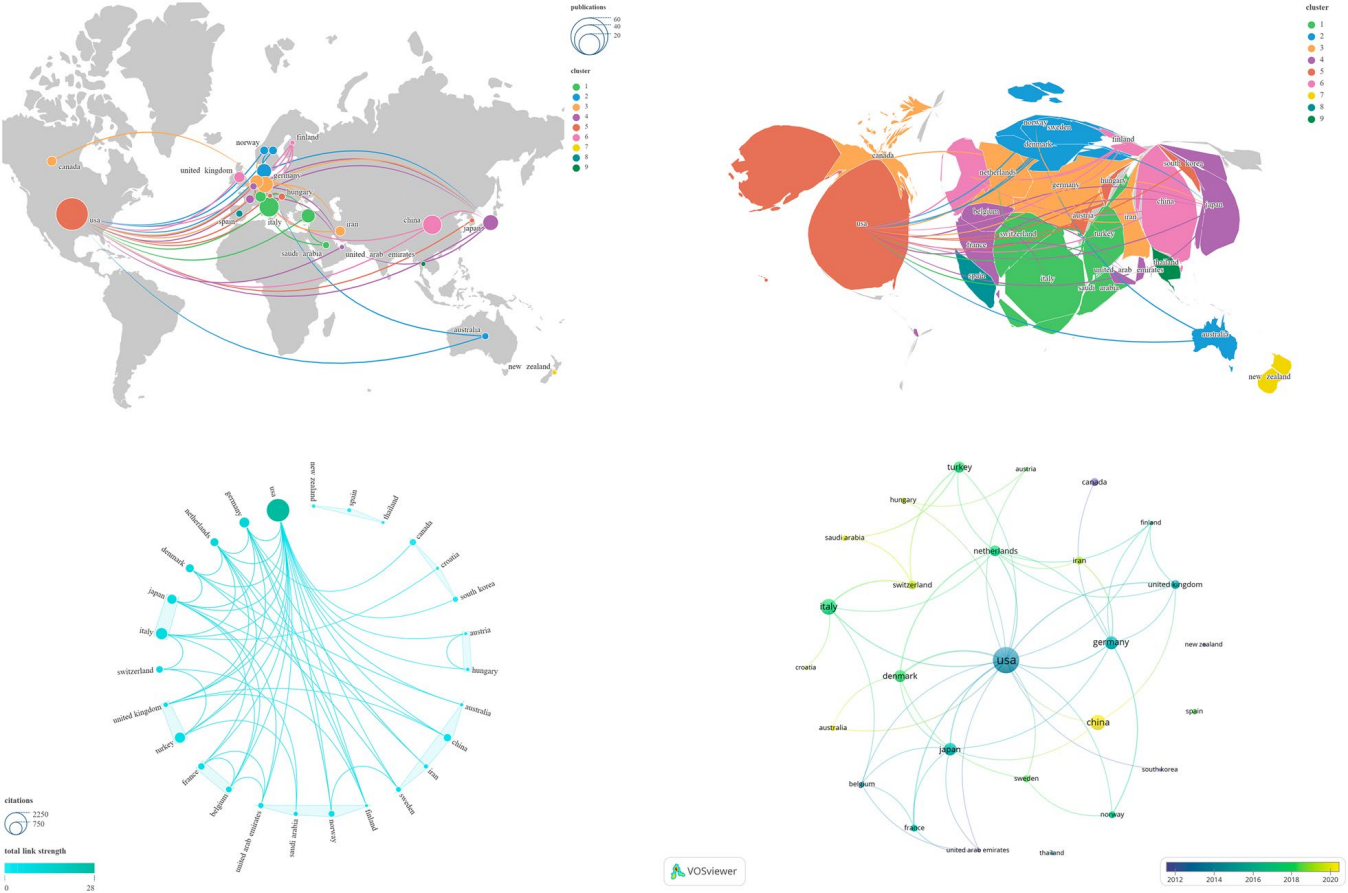
Visualization map illustrating Countries/regions contributions

We merged certain country/region names, such as England, North Ireland, Wales, and Scotland, into the overarching category of the United Kingdom. Subsequently, we identified all 27 countries/regions involved in this study. These were then subjected to a visualization analysis, grouping the countries/regions primarily into 11 clusters (Fig. 6). The results revealed that the country cooperation network centered around the United States exhibited the largest scale, encompassing 18 countries/regions. Table 3 provides detailed data for the top 10 countries/regions, sorted in descending order by NP. “Links” denotes the quantity of countries/regions involved in collaborative relationships. The countries/regions attaining the highest rankings in both NP and NC are the United States (*N* = 43, *N* = 2241) and Italy (*N* = 16, *N* = 578), respectively. The highest average citations were observed for TURKEY (*N* = 58.63), followed by USA (*N* = 52.12). The countries/regions with the highest Links and total link strength are identical, namely the United States (*N* = 18, *N* = 28) and Germany (*N* = 8, *N* = 11), respectively.

**Table 3:** Leading 10 countries/regions and institutions in terms of productivity

Rank	Countries/Regions	NP	NC	Total Link Strength	Links	Average citations	Institutions	NP	NC	Total Link Strength	Links	Average citations
1	USA	43	2241	28	18	52.12	CNR	7	334	26	11	47.71
2	ITALY	16	578	8	5	36.13	UNIV PADUA	8	332	30	9	41.5
3	CHINA	15	234	3	3	15.6	UNIV POLITECN MARCHE	4	127	19	8	31.75
4	GERMANY	11	435	11	8	39.55	UNIV COPENHAGEN	6	217	20	6	36.17
5	JAPAN	10	377	8	7	37.7	LEIDEN UNIV	4	56	19	6	14
6	DENMARK	9	280	9	5	31.11	HARVARD UNIV	5	249	17	3	49.8
7	TURKEY	8	469	6	4	58.63	AARHUS UNIV	6	170	16	3	28.33
8	NETHERLANDS	7	273	10	8	39	HACETTEPE UNIV	6	439	10	2	73.17
9	SWITZERLAND	5	182	7	3	36.4	UNIV CALIF LOS ANGELES	4	316	7	1	79
10	UNITED KINGDOM	5	77	6	5	15.4	CHONGQING MED UNIV	4	68	3	0	17

**Fig. 7:**
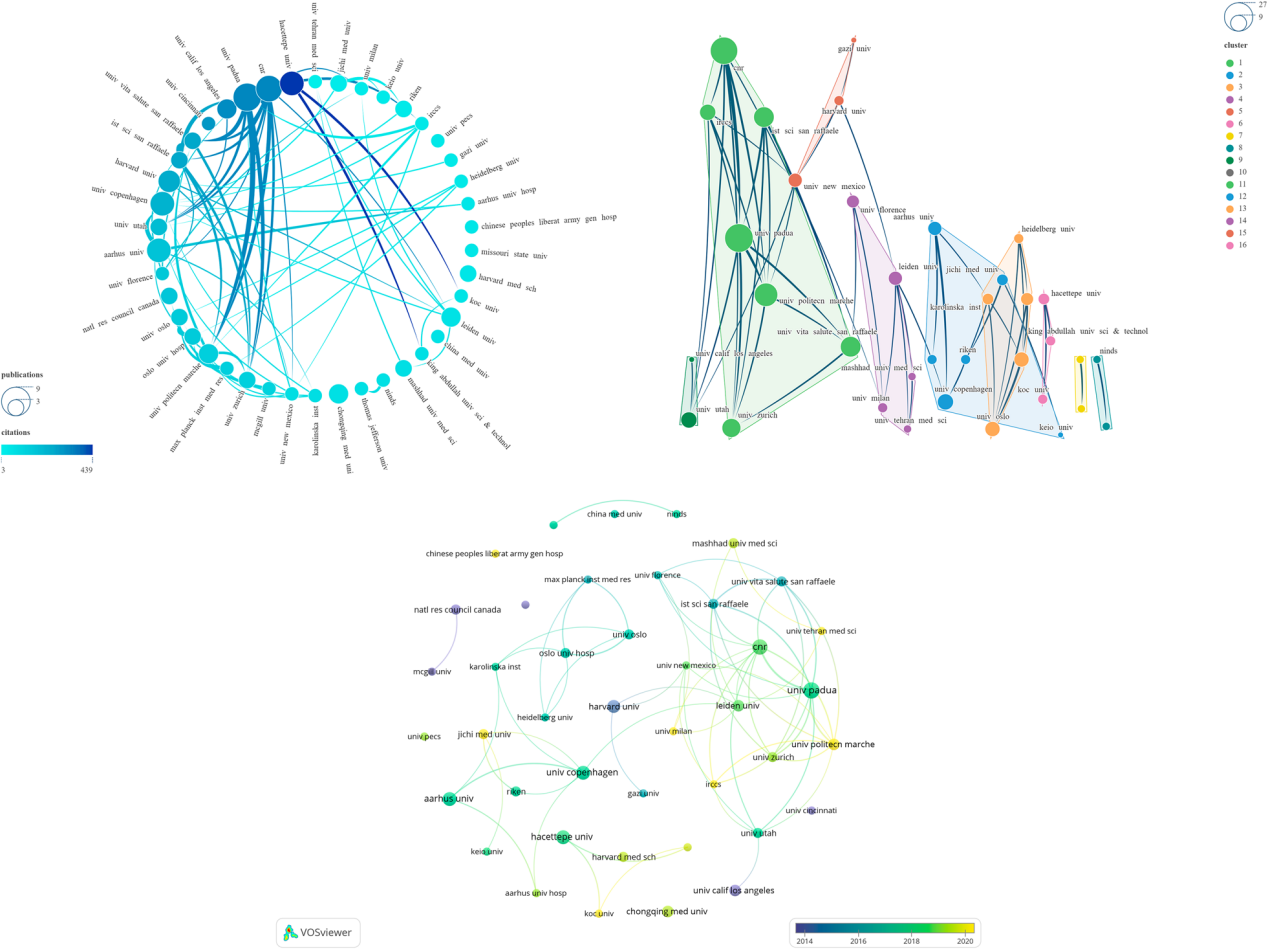
Visualization map illustrating Institutions contributions

Subsequently, we selected 42 institutions from the total of 220 institutions based on the criteria “Minimum number of documents of an article = 2” and conducted a visualization analysis on them (Fig. 7). The results revealed that these institutions were primarily divided into 16 clusters, with detailed data of the top 10 institutions, sorted in descending order by NP, listed in Table 3. The institution ranked first in NP is UNIV PADUA (*N* = 8), followed by CNR (*N* = 7). Regarding NC, the leading institution is HACETTEPE UNIV (*N* = 439), followed by CNR (*N* = 334). The institution ranked first in average citations is UNIV CALIF LOS ANGELES (*N* = 79), followed by HACETTEPE UNIV (*N* = 73.17). As for Total Link Strength, UNIV PADUA (*N* = 30) and CNR (*N* = 26) hold the top positions. In terms of Links, CNR (*N* = 11) ranks the highest, followed by UNIV PADUA (*N* = 9). It is noteworthy that CHONGQING MED UNIV demonstrates the lowest Links (*N* = 0) and Total Link Strength (*N* = 3) among the leading 10 institutions.

### Analysis of research categories

The literature within the field, categorized according to Web of Science Categories, spans various research directions. The most prominent categories garnering attention are Neurosciences (*N* = 69) and Clinical Neurology (*N* = 35).

### Keyword analysis

**Fig. 8:**
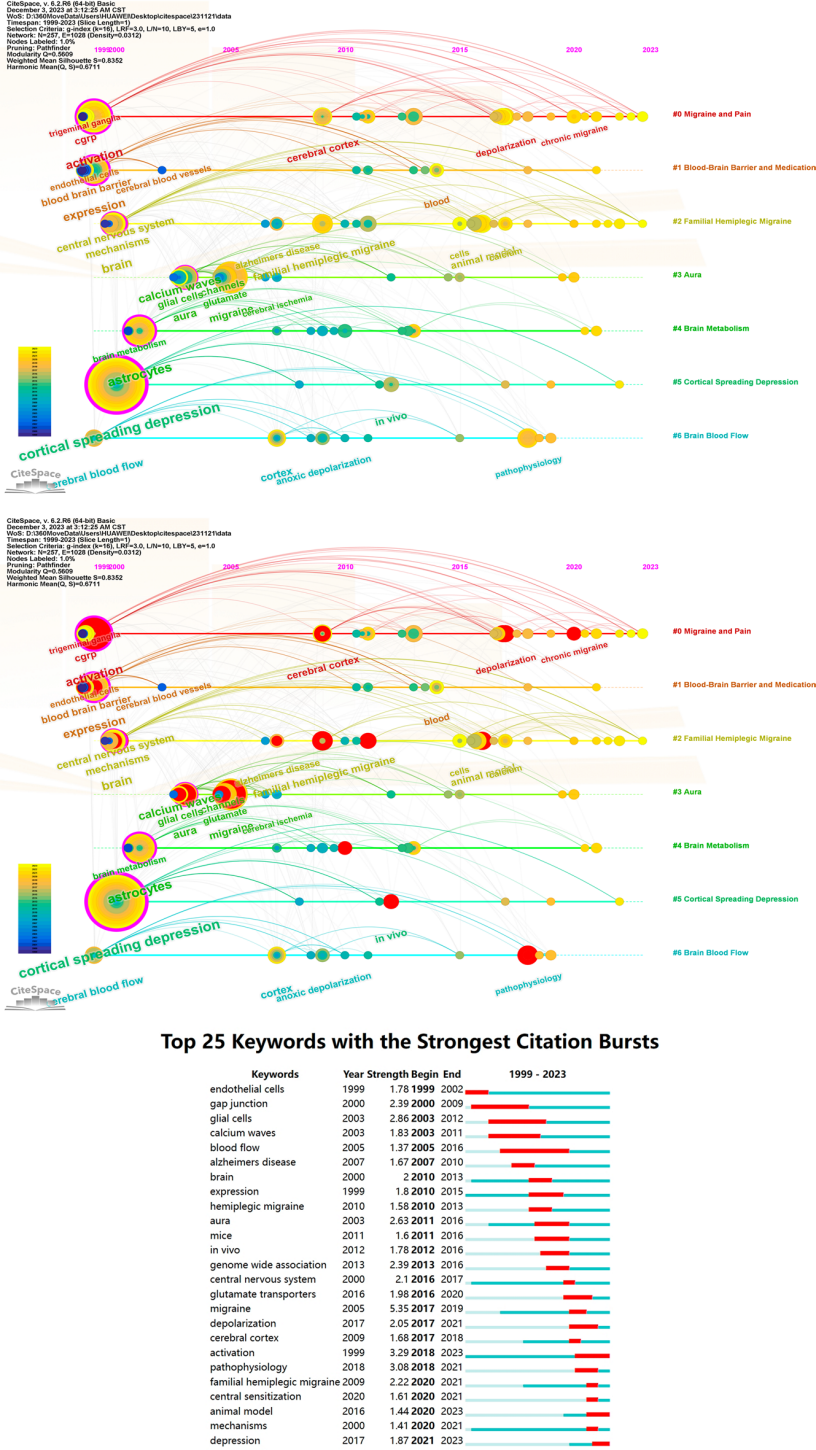
Visualization map illustrating Keyword clustering analysis by year and Keyword Burstiness analysis

Co-occurrence analysis is a common method in bibliometrics used to illustrate the frequency and patterns of research subjects, thereby revealing research hotspots [26]. Burstiness refers to objects that appear with a higher frequency within a certain time period, effectively reflecting the evolving trends of research hotspots within the field over time [27]. In total, 865 keywords were identified. We meticulously consolidated synonymous keywords, for example, merging terms like ‘calcitonin gene-related peptide,’ ‘calcitonin gene-related peptide (CGRP),’ and ‘gene-related peptide’ into a unified category labeled as ‘CGRP,’ ensuring precision in our keyword classification. Afterward, a refined selection process was employed by configuring the scale factor k = 16 in CiteSpace and utilizing the Pathfinder and Pruning Sliced Networks options in the pruning panel, resulted in the meticulous selection of 257 pivotal keywords for inclusion in our analysis. The assessed data is presented in Table 4 and Fig. 8. Notably, keywords such as ‘migraine,’ representing specific research domains, are not within the purview of our analysis. The most prevalent keywords observed are cortical spreading depression (*N* = 47), activation (*N* = 21), expression (*N* = 17), brain (*N* = 13), mechanisms (*N* = 13), central nervous system (*N* = 10), aura (*N* = 10), glutamate (*N* = 10), cerebral cortex (*N* = 8), familial hemiplegic migraine (*N* = 8), animal model (*N* = 8), pathophysiology (*N* = 8), alpha2 isoforms (*N* = 8), cgrp (*N* = 7) and cerebral blood flow (*N* = 7). The most prevalent neurotransmitters and signaling molecules identified are glutamate (*N* = 10) and CGRP (*N* = 7). The most frequently associated diseases and symptoms include alzheimers disease (*N* = 4), brain injury (*N* = 3), stroke (*N* = 2), and cerebral ischemia (*N* = 2). The most prevalent cell types identified are Neurons (*N* = 5), Glial Cells (*N* = 6), Endothelial Cells (*N* = 3), Smooth Muscle Cells (*N* = 2), and Satellite Glial Cells (*N* = 2). The most prevalent brain structures and regions identified include Cerebral Cortex (*N* = 8), Trigeminal Ganglia (*N* = 4), and Prefrontal Cortex (*N* = 2). Subsequently, we selected the log-likelihood ratio algorithm for clustering analysis, manually refined the cluster labels, and proceeded with visual analytics. The cluster map (Q = 0.5609, S = 0.8352) indicates satisfactory clustering quality, with Q > 0.3 and S > 0.5 [28]. The results indicate that these keywords were predominantly grouped into seven clusters, namely Cluster #0 Migraine and Pain, Cluster #1 Blood–Brain Barrier and Medication, Cluster #2 Familial Hemiplegic Migraine, Cluster #3 Aura, Cluster #4 Brain Metabolism, Cluster #5 Cortical Spreading Depression, and Cluster #6 Brain Blood Flow.

**Table 4:** Top 7 influential keywords and clusters in this research

Rank	Keywords	Count	Year	Centrality	ClusterID	Size	mean(Year)	LLR
1	CORTICAL SPREADING DEPRESSION	47	2000	0.51	0	38	2013	Satellite glial cell (8.79, 0.005); microglia (4.58, 0.05); dorsal pons (4.38, 0.05); infra-slow oscillations (4.38, 0.05); mechanosensitivity (4.38, 0.05)
2	ASTROCYTES	22	2001	0.34	1	36	2006	Endothelial cells (11.06, 0.001); smooth muscle cells (6.56, 0.05); protein (5.09, 0.05); intercellular adhesion molecule-1 (5.09, 0.05); long-term treatment (5.09, 0.05)
3	ACTIVATION	21	1999	0.2	2	34	2012	Subunit (7.32, 0.01); na,k atpase alpha 2 isoform (7.32, 0.01); nmda receptor (3.85, 0.05); mouse models (3.85, 0.05); mechanisms (3.72, 0.1)
4	MIGRAINE	20	2005	0.08	3	32	2007	Glial cells (7.44, 0.01); channel (4.22, 0.05); blood flow (4.22, 0.05); ion channels (3.85, 0.05); homeostasis (3.85, 0.05)
5	EXPRESSION	17	1999	0.24	4	28	2007	Brain energy metabolism (8.6, 0.005); glycogen (5.03, 0.05); tonabersat (4.29, 0.05); trigeminovascular (4.29, 0.05); partial inhibition (4.29, 0.05)
6	BRAIN	13	2000	0.18	5	23	2011	Adenosine (7.23, 0.01); glucose (3.77, 0.1); postprandial hypoglycemia (3.6, 0.1); adenosine receptors (3.6, 0.1); pathogenesis (3.6, 0.1)
7	MECHANISMS	13	2000	0.05	6	22	2013	(3–10): migraine (6.05, 0.05); serotonin (6.05, 0.05); trigeminal nucleus (6.05, 0.05); tanacetum parthenium (6.05, 0.05); xe 133 inhalation (6.05, 0.05)

Examining the temporal dynamics of keywords, we observed that around 1999–2010, all seven major clusters garnered significant attention. During this period, notable keywords included cortical spreading depression, activation, expression, brain, mechanisms, central nervous system, aura, glutamate, familial hemiplegic migraine, cerebral cortex, cerebral blood flow, cgrp. Between approximately 2010 and 2015, sustained attention was observed across all seven major clusters. Noteworthy keywords during this period included genome wide association, pain, mice, in vivo, atp, brain energy metabolism, blood, cells, astrocyte elevated gene 1, activity modifying protein 1, headache. Around 2015–2020, researchers gradually reduced their focus on Cluster #3 and Cluster #6. Noteworthy keywords during this period encompassed alpha2 isoforms, animal model, pathophysiology, glutamate transporters, depolarization, neuronal activity, depression, calcium channel. Between 2020 and 2023, Cluster #0 and Cluster #2 received significant attention from researchers. During this period, prominent keywords included nmda receptor, prefrontal cortex, neuropathic pain, stimulation, receptor, satellite glial cell and trigeminovascular system.

Subsequently, we conducted burstiness analysis in CiteSpace by setting γ = 0.4. Nodes highlighted with red circles indicate their burstiness. The keywords with the highest burstiness include: activation (*N* = 3.29), pathophysiology (*N* = 3.08), glial cells (*N* = 2.86), aura (*N* = 2.63), genome wide association (*N* = 2.39), and gap junction (*N* = 2.39). Keywords that continue to exhibit sustained burstiness include activation (*N* = 3.29), animal model (*N* = 1.44), and depression (*N* = 1.87).

### Reference analysis

**Fig. 9:**
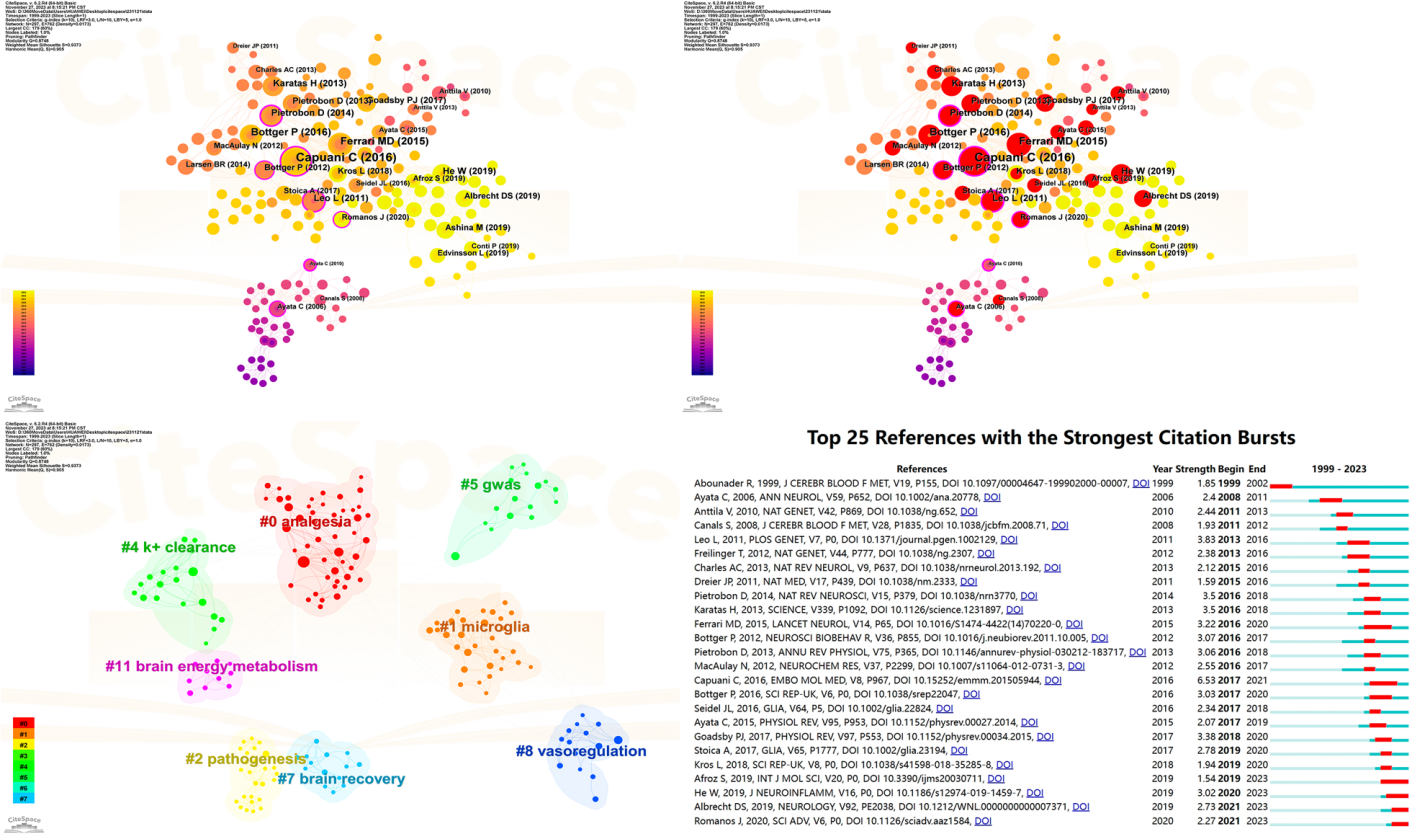
Visualization map illustrating Co-cited references clustering analysis by year and Co-cited references Burstiness analysis

We selected 297 references that met the criteria of a scale factor k = 10 out of a total of 6065 references and conducted visualization analysis on them. The assessed data is presented in Table 5 and Fig. 9. The most frequently cited document is “Defective glutamate and K + clearance by cortical astrocytes in familial hemiplegic migraine type 2” (*N* = 17), followed by “Migraine pathophysiology: lessons from mouse models and human genetics” (*N* = 11) and “Glutamate-system defects behind psychiatric manifestations in a familial hemiplegic migraine type 2 disease-mutation mouse model” (*N* = 10).” The clustering algorithm chosen was the log-likelihood ratio algorithm, leading to the generation of a clustering map (Q = 0.8748, S = 0.9373). The eight primary clusters comprise: Cluster #0 analgesia, Cluster #1 microglia, Cluster #2 pathogenesis, Cluster #4 K + clearance, Cluster #5 GWAS, Cluster #7 brain recovery, Cluster #8 vasoregulation, and Cluster #11 brain energy metabolism.

**Table 5:** The top 9 references and the top 8 clusters in This Research

Rank	Cited reference	Journals	Authors	Count	Brust	Year	Centrality	Cluster ID	Size	Mean (Year)	Label (LLR)
1	Defective glutamate and K + clearance by cortical astrocytes in familial hemiplegic migraine type 2	EMBO MOL MED	Capuani C	17	6.53	2016	0.24	0	49	2015	Analgesia (2.54, 0.5); nmda receptor (2.54, 0.5); alpha 2 na + (2.54, 0.5); waves (2.54, 0.5); nociception (2.54, 0.5)
2	Migraine pathophysiology: lessons from mouse models and human genetics	LANCET NEUROL	Ferrari MD	11	3.22	2015	0.02	1	31	2019	Microglia (8.91, 0.005); neurotransmitters (4.41, 0.05); postprandial hypoglycemia (4.41, 0.05); insulin sensitivity (4.41, 0.05); inflammation (4.41, 0.05)
3	Glutamate-system defects behind psychiatric manifestations in a familial hemiplegic migraine type 2 disease-mutation mouse model	SCI REP-UK	Bottger P	10	3.03	2016	0.06	2	26	2004	Pathogenesis (7.23, 0.01); astroglia (7.23, 0.01); pathophysiology (7.23, 0.01); diseases of the nervous system (7.23, 0.01); glia (4.51, 0.05)
4	Increased susceptibility to cortical spreading depression in the mouse model of familial hemiplegic migraine type 2	NAT REV NEUROSCI	Pietrobon D	8	3.5	2014	0.2	4	19	2014	k + clearance (3.34, 0.1); connexins kir4.1 inhibition by pkc (3.34, 0.1); na + -k + -atpase-mediated exit of 3 na + (3.34, 0.1); sodium imaging (3.34, 0.1); post-stimulatory undershoot (3.34, 0.1)
5	Chaos and commotion in the wake of cortical spreading depression and spreading depolarizations	J NEUROINFLAMM	He W	8	3.02	2019	0.02	5	16	2010	gwas (4.01, 0.05); cortisol (4.01, 0.05); glucagon (4.01, 0.05); brain metabolism (4.01, 0.05); insulin (4.01, 0.05)
6	Spreading depression triggers headache by activating neuronal Panx1 channels	PLOS GENET	Leo L	8	3.83	2011	0.34	7	14	2008	Brain recovery (6.61, 0.05); neural stem cells (6.61, 0.05); astrocyte (4.4, 0.05); spreading depression (4.4, 0.05); glial cells (3.91, 0.05)
7	Microglial NLRP3 inflammasome activation mediates IL-1β release and contributes to central sensitization in a recurrent nitroglycerin-induced migraine model	SCIENCE	Karatas H	8	3.5	2013	0.03	8	14	2017	Vasoregulation (4.08, 0.05); temporal characteristics (4.08, 0.05); treatment (4.08, 0.05); gut (4.08, 0.05); central sensitization (4.08, 0.05)
8	Pathophysiology of migraine: a disorder of sensory processing	PHYSIOL REV	Goadsby PJ	7	3.38	2017	0.07	11	10	2018	Brain energy metabolism (5.16, 0.05); mood disorders (5.16, 0.05); sleep (5.16, 0.05); astrocyte-neuron-lactate shuttle (5.16, 0.05); lactate (5.16, 0.05)
9	Pathophysiology of migraine	ANNU REV PHYSIOL	Pietrobon D	7	3.06	2013	0.02

Subsequently, employing a γ = 0.4 setting in CiteSpace, we conducted burstiness analysis. The findings revealed that the document exhibiting the highest burstiness is “Defective glutamate and K + clearance by cortical astrocytes in familial hemiplegic migraine type 2” (*N* = 6.53), followed by “Increased susceptibility to cortical spreading depression in the mouse model of familial hemiplegic migraine type 2” (*N* = 3.83), “Chaos and commotion in the wake of cortical spreading depression and spreading depolarizations” (*N* = 3.5), and “Spreading depression triggers headache by activating neuronal Panx1 channels” (*N* = 3.5). Currently, four publications continue to exhibit substantial burstiness: “CGRP induces differential regulation of cytokines from satellite glial cells in trigeminal ganglia and orofacial nociception” (*N* = 1.54), “Microglial NLRP3 inflammasome activation mediates IL-1β release and contributes to central sensitization in a recurrent nitroglycerin-induced migraine model” (*N* = 3.02), “Imaging of neuroinflammation in migraine with aura: A [11C] PBR28 PET/MRI study” (*N* = 2.73), and “Astrocyte dysfunction increases cortical dendritic excitability and promotes cranial pain in familial migraine” (*N* = 2.27).

## Discussion

### General information

In accordance with the temporal evolution of relevant literature in this field, we observed a consistent upward trajectory in the overall quantity of publications. Notably, over the past decade, the number of publications has significantly surpassed historical figures, with four years registering a count equal to or exceeding 10, all occurring post-2016. Given our literature inclusion cutoff date of November 21, 2023, we acknowledge that our analysis may not fully capture the publication landscape for the current year. Nevertheless, based on the observed trends (Fig. 2), we have reasonable grounds to anticipate sustained high activity in the field throughout 2023.

In the journal analysis aspect, CEPHALALGIA ranks first in NP, g-index, h-index, and total link strength, and it also holds impressive rankings in other indicators. Therefore, we believe CEPHALALGIA has exerted the greatest influence. JOURNAL OF HEADACHE AND PAIN consistently holds a leading position in multiple analyzed metrics, underscoring its undeniable influence in the field. The highest NC and Average Citations associated with JOURNAL OF NEUROSCIENCE indicate the superior quality of its published works, widely recognized within the scholarly community. Considering the total link strength, JOURNAL OF CEREBRAL BLOOD FLOW AND METABOLISM appears to warrant strengthened collaborative relationships. The annual publication trends further highlight the commendable growth and dominant position of CEPHALALGIA in the field in recent years. The evolving dynamics of journal publications over time suggest that JOURNAL OF HEADACHE AND PAIN and Neurobiology of Disease may be worth noting in this field recently (Fig. 3 and Table 1). Figure 4 unveils primary citation pathways, providing valuable insights for novice researchers embarking on studies in this field.

In the realm of author analysis, considering all encompassed metrics, we assert that PIETROBON D and DALKARA T wield significant influence in this field. Notably, EREN-KOCAK E, with a relatively lower NP count (*N* = 4), attains the third-highest NC (*N* = 405) and the highest Average Citations (*N* = 101.25), indicative of widespread recognition for the quality of their publications. The total link strength indicates that all eight scholars maintain close collaborative relationships with fellow specialists in the domain. The yearly variation in authors’ publication output reveal that PIETROBON D and CONTI F have exerted sustained influence in the domain. Furthermore, the evolving dynamics of journal publications over time suggest that CONTI F, among others, may be emerging as noteworthy and promising contributors in the field (Fig. 5 and Table 2).

The analysis of regions and countries reveals the most intricate and expansive collaboration network between Europe and North America, centered around the United States. The United States exhibits overwhelming dominance, boasting the highest number of links, total link strength, NP, and NC, thereby establishing extensive connections across regions. Italy, with its second-ranking NP and NC, coupled with rankings in other pertinent metrics, is deemed to exert substantial influence in this field. TURKEY holds the third position in NC and boasts the highest Average Citations, indicating that its published works are of high quality and widely recognized. While CHINA holds the third position in NP, considering its NC, Average Citations, and Total Link Strength, we suggest there is room for improvement in the quality of its publications, and fostering collaborative relationships could be beneficial. The evolving trends in country/region publication dynamics over time reveal recent vibrancy in the contributions of countries/regions such as CHINA in this field (Fig. 6 and Table 3).

Considering the outstanding performance of UNIV PADUA and CNR across various rankings, we unequivocally identify them as institutions wielding significant influence in this field. Among these, HACETTEPE UNIV boasts the highest NC and secures the second position in terms of Average Citations. UNIV CALIF LOS ANGELES achieves the highest Average Citations with a relatively lower NP. Hence, we posit that publications affiliated with HACETTEPE UNIV and UNIV CALIF LOS ANGELES demonstrate a higher quality. Notably, CHONGQING MED UNIV appears to benefit from strengthening collaborative efforts with other institutions. The annual variation in institutional publication output suggests that institutions such as UNIV POLITECN MARCHE, JICHI MED UNIV, and IRCCS may be noteworthy in this field recently (Fig. 7 and Table 3).

### Knowledge base

**Fig. 10:**
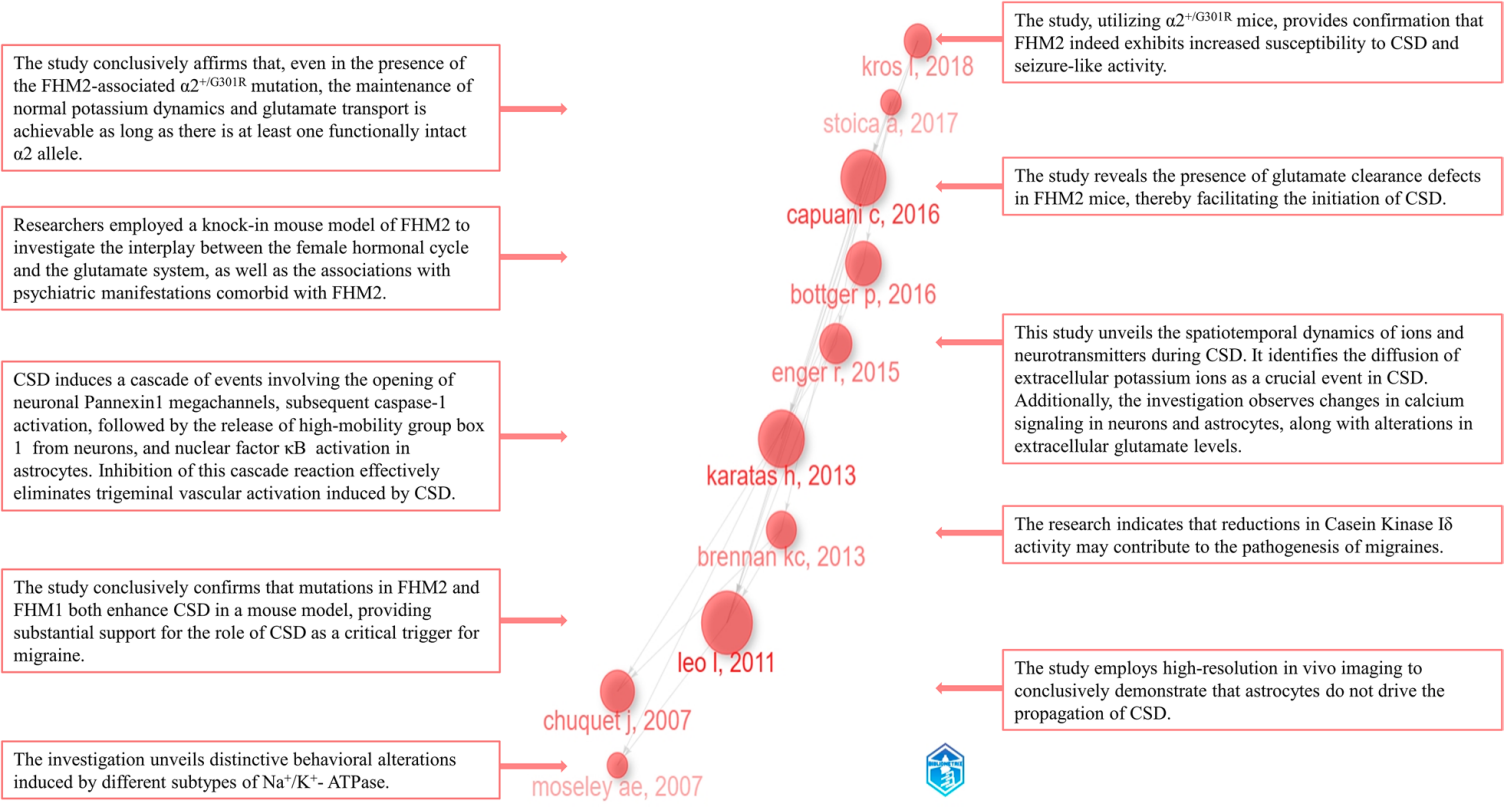
Principal contents of the top 10 milestone publications

Analyzing and interpreting pivotal publications, along with the results of keyword and reference analysis in this field, allows for a more in-depth understanding of its historical development trajectory. This process proves beneficial in gaining insights into both the past and present aspects of the field. We employed historiographic analysis using the R package ‘bibliometrix’ to identify and visually analyze 10 milestone publications within this field. Subsequently, we annotated the primary content and key information of these publications based on the original images, ultimately presented in Fig. 10. Combining the primary information from the 10 identified publications, we observed that all studies primarily focused on CSD, Na + /K + -ATPase, or FHM2. The majority of these investigations involved animal experiments, predominantly conducted in mouse models. A study revealed an increased susceptibility to CSD in FHM2 caused by a mutation in the α2 subunit of Na + /K + -ATPase. This research highlights the interrelation among these factors, laying a robust foundation for subsequent related studies [9]. Researchers predominantly focused on calcium waves associated [29], Casein kinase Iδ [30], PANX1 channels [31], dynamics of ionic shifts [32], the glutamate system [33, 34], K + clearance [34, 35], and migraine-related epileptiform activity [36]. One publication delineates the association between different subtypes of Na + /K + -ATPase and a spectrum of behavioral alterations [37]. In addition to the milestone publications mentioned above, research in this domain has also delved into numerous other facets. Several publications provide comprehensive summaries of the glial–neuronal interaction in migraine [20, 38] and the role of dysfunctional astrocytes [39]. Researchers have focused on different phases of migraine, including the aura phase [40] and the interictal phase [41]. The investigated subjects encompass mitochondrial function [42], insulin-like growth factor-1 [43], the CGRP system [44, 45], brain glycogen [46], 5-hydroxytryptamine receptor subtypes [47], and Na + /HCO3-cotransporter [48]. The cells, tissues, and sites under investigation include meningeal cells [49], neocortex [50], occipital regions [41], trigeminal ganglia, and brain vessels [45]. In addition, topics such as sleep [46], inflammation [51], and female-related factors [41] have garnered attention from researchers.

The most common keywords highlight the primary focus of researchers in this field, emphasizing the pathophysiological mechanisms of different types of migraines. Researchers place a significant emphasis on animal studies, evaluating the impact on the blood–brain barrier and hemodynamics in the central nervous system. Their attention spans across various genes and proteins, the status of multiple cell types, and signal transduction. In addition, there is a notable interest in CSD. In the period around 1999–2010, researchers prominently focused on migraines with aura and FHM, underscoring the pivotal role of CSD and placing emphasis on observing hemodynamic changes. Investigations delved into the cellular and molecular aspects within the brain and cortex, exploring relevant mechanisms. Simultaneously, glutamate and CGRP gained increasing attention as crucial factors in the migraine process. In the period around 2010–2015, researchers developed a keen interest in ATP and brain energy metabolism. The application of genome-wide association techniques saw a gradual increase over time. Through predominantly in vivo experiments using mice, investigations were conducted into astrocyte elevated gene 1 and activity-modifying protein 1. Simultaneously, researchers focused on symptoms such as headache and pain. From approximately 2015 to 2020, new focal points emerged, including alpha2 isoforms, glutamate transporters, and calcium channels. Mechanistic explorations regarding depolarization, neuronal activity, and depression saw an increasing trend. The crucial role of animal models in pathophysiological research was further acknowledged during this period. As of 2023, research pertaining to satellite glial cells has captured the attention of scholars. In addition, researchers have turned their focus towards studying the prefrontal cortex, trigeminovascular system, and other organizational structures. Emerging research priorities include neuropathic pain, stimulation, and NMDA receptor-related investigations. The clustering of keywords underscores the diverse directions of research, encompassing migraine symptoms, mechanisms, organizational structures, influencing factors, and various types of migraines. The primary symptoms of migraine, particularly pain, have consistently been the focus of extensive research. FHM and migraine with aura stand out as two prominent subtypes garnering substantial attention from researchers. Notably, CSD plays a pivotal role in these migraine types, attracting significant interest. Researchers have also delved into the impacts of the blood–brain barrier (BBB) and the effects of medications. In addition, investigations into brain metabolism and blood flow have been pivotal areas of study (Fig. 8 and Table 4).

The prevalence of citations highlights researchers’ focus on investigating the pathophysiological mechanisms of migraine [52–54]. CSD remains a focal point of scholarly attention in this field [31, 55]. “Three studies, employing murine models, focused on Glutamate system defects, K + clearance, and CSD in association with astrocytes in FHM [9, 33, 34]. One publication predominantly investigated the inflammatory response of the Microglial NLRP3 inflammasome in central sensitization of chronic migraine [56]. In addition to the nine most frequently cited references encompassing prominent research areas, our comprehensive review of other references reveals that researchers in this field have also placed significant emphasis on topics such as aura, neuroimaging, the trigeminal vascular system, Genome-Wide Association Studies, CGRP, and satellite glial cells [10, 57–59]. Cluster analysis of the references underscores diverse research domains, with researchers concentrating on specific pathophysiological mechanisms, notably vasoregulation, brain recovery, and brain energy metabolism. The critical role of K + clearance in the sustained functioning of the nervous system has also garnered considerable attention among researchers. Researchers have directed their attention towards the application of genetic technologies, particularly Genome-Wide Association Studies, and various cell types, with a focus on Microglia. In addition, analgesia has consistently served as a pivotal metric for assessing the efficacy of migraine treatment (Fig. 9 and Table 5).

### Research highlights

The results of keyword and reference clustering analysis highlight the primary areas of interest for researchers. Combining these findings with our understanding of the field, we succinctly summarize several major research hotspots, including CSD, Aura and Brain Blood Flow, FHM, BBB and Medication, Brain Metabolism, and the Brain’s Glymphatic System (BGS).

### CSD

CSD is a distinctive abnormal phenomenon. It initiates with widespread depolarization of cortical neurons and glial cells, followed by a gradual reduction in activity. It propagates at a speed of approximately 3–4 mm/min, potentially reaching the parietal and/or temporal lobes [60, 61]. The widespread depolarization signifies a pronounced alteration in the concentration of various ions both inside and outside the cells. These changes result in cell swelling, significantly impacting the composition of the extracellular space. Notably, K + and glutamate receptors, particularly N-methyl-D-aspartate receptors, play crucial roles in the initiation of CSD [62, 63]. During the CSD process, various substances, including ions, vasoactive substances, inflammatory mediators, and neurotransmitters, are intricately linked to vasodilation, plasma protein extravasation, mast cell degranulation, and other cellular processes. These interactions may potentially trigger migraines through pathways such as activating the trigeminovascular system and inducing neuroinflammation [64]. Current research evidence indicates that CSD might serve as a trigger for auras [60]. Furthermore, CSD has been shown to induce changes in blood vessel diameter [65]. Consistent with the higher prevalence of migraines in females compared to males, female mice exhibit a higher susceptibility to CSD than their male counterparts [66]. Beyond its association with migraines, CSD has also been implicated in other conditions, such as stroke and head trauma [67].

Currently, the role of astrocytes in CSD remains contentious, but some evidence suggests a connection between the two. Ca2 + waves in astrocytes occur almost simultaneously with depolarization of neurons during CSD [50]. Certain studies propose that astrocytes might be involved in the initiation of CSD [9, 68]. In addition, astrocyte networks likely play a crucial role in the regulation of K + and glutamate during CSD and the propagation of CSD [67, 69].

### Aura and brain blood flow

Approximately one-fourth to one-third of migraine sufferers experience an aura before, during, or even after a migraine attack [64]. An aura is a transient and reversible focal neurological symptoms, involving aspects such as vision, sensation, speech, movement, and brainstem functions. Typically, auras develop gradually over 5–20 min and gradually resolve within 60 min [2]. The current mechanisms of aura remain elusive, with the prevailing view suggesting a crucial role for CSD in its occurrence. In alignment with the abundance of vascular-related keywords present in keyword cluster #3, the initial association of CSD is with hyperemia. This hyperemia, appearing approximately 15 s after CSD onset, can persist for up to 3 min. Subsequently, hyperemia gives way to oligemia. The duration of this oligemia can extend up to 1 h or even longer [70]. Such oligemia may impact neurons and glial cells in various brain regions by inducing local tissue ischemia and hypoxia, consequently triggering the onset of an aura. Conversely, ischemia or hypoxia can also serve as triggering conditions for CSD [71]. “In addition, the drastic changes in intracellular and extracellular ion concentrations and the release of substances induced by CSD may also contribute to the aura of migraines. Presently, multiple imaging studies provide evidence that CSD and the associated hemodynamic changes are correlated with auras [64]. The controversy persists regarding whether auras trigger migraine attacks, with a hypothesis suggesting that pain and auras may represent two parallel phenomena [72]. Moreover, various substances produced during CSD, including vasoactive agents, can induce changes in cerebral blood flow through their impact on brain vasculature during the CSD process [64].

### FHM

FHM is an exceptionally rare form of monogenic migraines, characterized by autosomal dominant transmission caused by pathogenic mutations in a single gene. FHM is notably distinguished from migraines with aura by the obligatory inclusion of motor symptoms during its aura manifestation, with the aura can even extend for several dozen hours. In addition, a crucial criterion for FHM diagnosis is that the patient must have at least one first-degree or second-degree relative diagnosed with FHM; otherwise, it is classified as sporadic Hemiplegic Migraine [2]. Mutations in ion transport genes CACNA1A, ATP1A2, and SCN1A respectively impact the encoding of Cav2.1 channels, α2 Na + /K + -ATPase, and Cav1.1 channels, leading to three distinct forms of FHM: FHM1, FHM2, and FHM3[73]. Based on our analysis, it is evident that the focal point of research in this field revolves around ATP1A2-related FHM2. In adult astrocytes, Na + /K + -ATPase expressed on the cell surface maintains K + balance in the synaptic cleft through K + clearance. This mechanism restricts neuronal excitability and sustains the transmembrane Na + concentration gradient, facilitating the reuptake of glutamate. In the context of FHM2, mutations lead to dysfunctional α2 Na + /K + -ATPase, potentially impairing the reuptake of K + and glutamate by astrocytes. Consequently, this dysfunction may contribute to a delayed recovery of neuronal excitability [73].

### BBB and medication

The BBB primarily consists of brain microvascular endothelial cells, the basement membrane surrounding endothelial cells, pericytes, and the foot processes of astrocytes. By restricting the passage of certain molecules, the BBB serves as a barrier between the systemic circulation and the central nervous system, the BBB plays a crucial role in protecting the brain and maintaining its homeostasis [74]. Astrocytes play a crucial role in both the homeostasis and homeostatic imbalance of the BBB. Astrocytes contribute to the maintenance of BBB stability through various substances, such as Sonic hedgehog [75] and Sphingosine 1 [76]. In addition, their influence on smooth muscle function impacts the regulation of cerebral microvascular blood flow associated with the BBB [77]. In various pathological conditions, astrocytes can undergo transformation into reactive astrocytes. These reactive astrocytes also play a crucial role in the BBB, such as contributing to its repair following neurological diseases [78, 79]. Due to the existence of the BBB, drug components that need to exert their effects in the brain must traverse this barrier. The primary mechanisms include diffusion, carrier-mediated transport, endocytosis, among others. However, the BBB is influenced by various factors such as inflammation, neuropeptides, and glutamate, leading to alterations in its permeability and other aspects [80]. which has been an unavoidable focus in migraine drug research.

### Brain metabolism

According to the content of Cluster #4 “Brain Metabolism” in the keywords analysis, researchers in this field focus on various subjects, including SLC4A4, nitric oxide, pyruvate carboxylase, coenzyme Q10, among others. However, human metabolism is comprised of thousands of biochemical reactions. Confronted with the vast and intricate network composed of metabolic byproducts, associated proteins, and the genome resulting from various metabolic processes, elucidating the mechanisms within this complex system is undoubtedly a formidable task. Omic technologies, as a method for discerning key targets and analyzing vast datasets, offer a valuable tool to alleviate the challenges faced by researchers [81]. Human Genome-Scale Metabolic Models, constructed based on existing literature, represent a widely utilized computational framework in metabolic research. These models primarily focus on metabolic processes and encompass virtually all substances involved in metabolic reactions, contributing significantly to advancements in the field [82]. Genome-Scale Human Brain Modeling is a computational model primarily focused on the dynamics of the human brain, playing a significant role in the field [83]. The application of these advanced methods will aid researchers in elucidating the intricate relationships within metabolic networks and contribute to the generation of novel insights.

### BGS

The BGS, distinct from the classical lymphatic circulation system, has garnered increasing attention recently [84]. It is, in fact, a complex network of perivascular spaces containing a significant volume of cerebrospinal fluid and interstitial fluid, surrounding brain vessels and communicating with the subarachnoid space. This network functions as the brain’s lymphatic circulation, maintaining homeostasis by clearing metabolic waste and various solutes from the brain [85]. The water transporter Aquaporin 4, located on the foot-like protrusions of astrocyte cells, plays a crucial role in the process of cerebrospinal fluid and interstitial fluid entering cervical lymphatics [86]. Neuroinflammation, CSD, and excessive CGRP are three mechanisms that have garnered widespread attention in the pathogenesis of migraine and aura. The BGS may exert influence on the aforementioned three mechanisms by regulating pro-inflammatory substances, CGRP, and relevant ion concentrations, thus significantly contributing to the processes of migraine and aura [87]. However, the significant role of the BGS in migraine research has not received adequate attention. We posit that in the future, the BGS may emerge as a focal point of investigation in this field.

### Prospects for the future

Based on our analysis of relevant literature in the field, along with burstiness analysis of keywords and the results of keyword clustering, we offer a reasonable outlook for the future development of this field. We anticipate that researchers will continue to explore various pathophysiological mechanisms of migraine through animal models, with a primary focus on migraines with aura and FHM. There will be ongoing attention to diverse pain symptoms associated with migraines, using them as metrics to evaluate treatment efficacy. Emphasis will be placed on investigating cellular and tissue structures such as satellite glial cells and the trigeminal ganglion. The application of imaging techniques and sustained monitoring of inflammation, central sensitization, and CGRP will remain crucial. Further in-depth exploration of the crucial role played by astrocytes in migraine is imperative.

### Limitations

We must acknowledge certain limitations in our study. While Web of Science is extensively used as a literature retrieval database, it may result in the omission of some relevant studies. We have strived to standardize inclusion and exclusion criteria to the best of our ability, but biases may still be present. In addition, the exclusion of studies published in languages other than English may underestimate the impact of non-English written papers.

## Conclusion

In summary, we conducted a pioneering bibliometric analysis aimed at revealing the pivotal role of astrocytes in migraines, encompassing visualization analysis of journals, authors, countries/regions, institutions, keywords, and references. Based on the analysis findings, we synthesized the current foundational knowledge, inferred future trends, and provided an overview of current research hotspots. We posit that researchers should continue to prioritize investigations into aura-associated migraines, FHM, and the crucial CGRP system. Leveraging advanced observational techniques, particularly in imaging, is crucial. Attention should be directed towards cellular and tissue structures such as small glial cells and trigeminal ganglia, as well as mechanisms like inflammation and central sensitization. Furthermore, a concerted effort should persist in conducting extensive pathophysiological studies based on animal models to accumulate a robust body of high-quality evidence.

### Guidance for researchers

1. Emphasize the Standardization of Animal Models: In the realm of migraine research, where a myriad of model-establishment methods exists, meticulous quantification is paramount. For techniques involving drug injections, precision in timing, accurate positioning, and even injection angles should be quantified with greater attention to detail.
2. Enhance Objective and Clear Interpretation of Research Findings: Strive for an elevated level of objectivity and clarity when interpreting research results. A meticulous and unambiguous presentation of findings is essential for advancing the field.
3. Prioritize the Reproducibility of Experiments: Reinforce the significance of experiment reproducibility. Ensuring that experimental procedures can be replicated with precision contributes to the robustness and reliability of scientific outcomes.
4. Underscore the Importance of Genomic Data Sharing: Recognize the value of sharing genomic data. Facilitating open access to genomic datasets contributes to the collaborative advancement of knowledge in the field.
5. Strengthen Cross-Regional Collaboration: Foster collaboration across regions. Recognizing the potential for diverse perspectives and expertise in different geographical areas can lead to more comprehensive and impactful research outcomes.

## Data Availability

The datasets used and/or analysed during the current study are available from the corresponding author on reasonable request.
